# Editorial: The Serpin Family in the Cardiovascular System

**DOI:** 10.3389/fcvm.2021.821490

**Published:** 2022-02-02

**Authors:** Marie-Christine Bouton, Javier Corral, Alexandra R. Lucas

**Affiliations:** ^1^LVTS, INSERM, U1148, Université de Paris, X. Bichat Hospital, Paris, France; ^2^Servicio de Hematología y Oncología Médica, Hospital Universitario Morales Meseguer, Centro Regional de Hemodonación, Universidad de Murcia Campus Mare Nostrum, IMIB, CIBERER, Murcia, Spain; ^3^Center for Personalized Diagnostics and Center for Immunotherapy, Vaccines and Virotherapy, The Biodesign Institute, Arizona State University, Tempe, AZ, United States

**Keywords:** serpins, thrombosis, fibrinolysis, inflammation, therapy

In this special Research Topic, five original research articles and eight reviews are published under the title of ≪ Serpins in the cardiovascular system ≫. This unique Research Topic on serpins (SERine Protease INhibitors) provides an overview of the recent advances on the role of anticoagulant and antifibrinolytic serpins, as well as viral serpins or non-inhibitory serpins, in the cardiovascular system. There is a wealth of knowledge to be discovered in these intricate regulators, the serpins, that represent up to 2–10% of circulating blood proteins and control many physiologic processes (1–4) (Figure 1).

**Figure 1:**
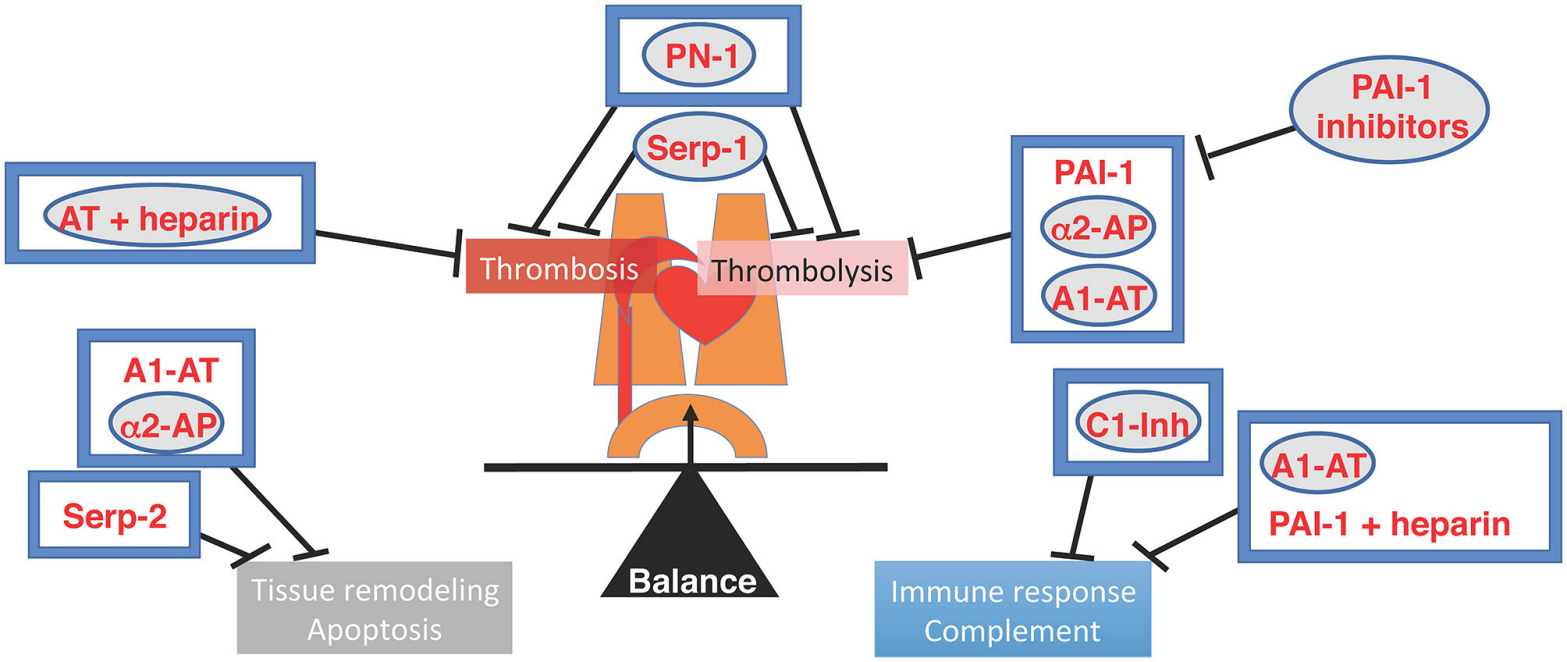
Illustration of the Cardiovascular system and the balancing of Serine proteases and SERPIN interactions. Native regulatory pathways are in boxes. Therapeutic serpins are in circles. Serpins reviewed in this special issue have a gray backdrop. AT, Antithrombin; A1-AT, alpha1-antitrypsin; α2 AP, alpha2-antiplasmin; C1-Inh, C1-inhibitor; PAI-1, plasminogen activator type-1; PN-1, protease nexin-1.

A mini review by Shu et al. shares the recent resolution of structural studies of angiotensinogen, the unique non-inhibitory serpin evoked in this Research Topic. The authors describe the crystallographic studies explaining the interaction of human angiotensinogen with human renin and hence the mechanism of angiotensin release.

The two antifibrinolytic serpins, Plasminogen activator inhibitor type-1 (PAI-1) and Alpha2-Antiplasmin (α2-AP), were thoroughly described in this topic. PAI-1 was described in two complementary reviews. One was written by Sillen and Declerck who provide a precise review of its structure/function and describe diverse approaches for the development of PAI-1 inhibitors. The other study, written by Morrow et al., details the role of PAI-1 in a variety of pathophysiological conditions, in particular in thrombo-inflammation and in the metabolic syndrome. An overview of α2-AP in thrombosis and cardiovascular diseases was reported by Singh et al. The authors describe how targeting this fast-reacting inhibitor of plasmin may provide potential therapeutic opportunities. Protease nexin-1 (PN-1), another antifibrinolytic serpin described in this Research Topic, was detailed by Madjene et al. In contrast to the two previous serpins depicted above, PN-1 displays both anticoagulant and antifibrinolytic properties, which makes this serpin special. The authors summarized the many functions attributed to this enigmatic serpin in health and cardiovascular diseases.

The potential use of serpins as therapeutic targets for bleeding or thrombotic disorders was reviewed by Bianchini et al. who summarize the drawbacks and advantages of targeting specific serpins involved in the finely tuned balance between procoagulant and anticoagulant systems.

The use of serpins for therapeutic is a recent growing area of clinical investigation. Such an idea of therapeutic serpins was assessed by Maas and De Maat who describe the different approaches of engineering serpins to optimize their potential therapeutic relevance. A complementary review on serpin roles in fibrinolysis and inflammation and their potential therapeutic applications was written by Yaron et al. It provides an overview of a highly potent class of serpin virus-derived biologics. One such serpin, Serp-1, is a 55 kDa secreted myxomavirus serpin that, like PN-1, can bind and inhibit proteases in both the thrombotic and thrombolytic cascades targeting sites of serine protease activation (1, 5–7) Guo et al. This serpin has been tested in a wide range of animal models of inflammatory disorders, and it also appears successful in a small phase-2 clinical trial in unstable coronary patients receiving stent implants. This work, together with mammalian serpins already used in clinic for genetic disorders such as C1-inhibitor (C1-inh) (8) and α1-antitrypsin (A1-AT), provides a basis for using serpins to target dysfunctional protease pathways. The authors discuss the potential use of serpins for treating COVID-19 since a non-negligible number of serine proteases such as thrombin, urokinase-type plasminogen activator (uPA) and complement, the main targets of serpins, are critically involved in SARS-Cov-2 infection. Indeed, this viral serpin, Serp-1, as well as antithrombin (AT), A1-AT, and C1-inh are being discussed and investigated as potential therapeutics for use in acute respiratory distress syndrome (ARDS), cytokine storm, and microthrombotic coagulopathy in COVID-19 (8–10). As serpins are inhibitors, these naturally evolved regulatory inhibitors of proteases have outstanding potential as therapeutics in severe viral infections complicated by aggressive inflammatory and coagulopathic disorders where treatment remains very limited.

In this topic, we have also three excellent examples of how genetic variants of serpins help to understand the impact of serine protease inhibitors in cardiovascular diseases. One of these variants, named the AT variant p.Leu131Phe (AT Budapest 3), is classified among the type II Heparin-binding site (HBS) deficiencies as it does not severely reduce the secretion to the plasma but impairs the activation of AT by heparin (11). In this topic, Natae et al. have evaluated this variant in the Hungarian Roma population, where the p.Leu131Phe variant has a founder effect and reaches a high frequency (1%), aiming to find genetic-environmental interactions that might explain the increased susceptibility to venous thrombosis of this population. This study finds five positive and significant genetic and environmental interactions that play a role in venous thrombosis and should be considered for the assessment of thrombotic disease susceptibility. This study was fully complemented by the manuscript of Bereczky et al. who have done a brilliant archaeogenetic analysis that revealed the age and evolution of the p.Leu131Phe variant, a 400-year-old founder mutation. The bonus of this study was the analysis of the role of this and other type II HBS variants on the risk of arterial thrombosis. Interestingly, the study of Bereczky et al., found consistent evidence to consider AT type II HBS deficiencies as a risk factor not only for venous but also for arterial thromboembolism, especially in selected populations as young patients without advanced atherosclerosis, data supported by other studies (12, 13). This finding encourages the diagnosis of these genetic variants, particularly in populations where they could be frequent (e.g., in Hungary, Spain, and Finland), and would be useful for the management of carriers, as an early diagnosis might warned carriers to avoid any modifiable cardiovascular risk factors. Finally, the study of Hamada et al. is another example of the therapeutic expectations of “a la carte” recombinant serpins with new functions as mentioned by Maas and De Maat. Here, the authors explore A1-AT, an elastase inhibitor whose deficiency is involved in pulmonary emphysema, to create a strong and specific FXIa inhibitor that might be used as a new anticoagulant. By using elegant methods, Hamada et al. have engineered a recombinant A1-AT variant AAT-RC-2 as a specific FXIa inhibitor whose potential clinical benefit must be evaluated in further studies.

We believe that this original topic on serpins will allow readers to appreciate the extraordinary contribution of numerous proteins of this superfamily in the cardiovascular system and show how attractive targets they could be for drug discovery.

## Author Contributions

All authors listed have made a substantial, direct, and intellectual contribution to the work and approved it for publication.

## Funding

Financial supports from ISCIII & FEDER (PI21/00174) to JC are gratefully acknowledged.

## Conflict of Interest

The authors declare that the research was conducted in the absence of any commercial or financial relationships that could be construed as a potential conflict of interest.

## Publisher's Note

All claims expressed in this article are solely those of the authors and do not necessarily represent those of their affiliated organizations, or those of the publisher, the editors and the reviewers. Any product that may be evaluated in this article, or claim that may be made by its manufacturer, is not guaranteed or endorsed by the publisher.
